# Genetic Influences on Metabolite Levels: A Comparison across Metabolomic Platforms

**DOI:** 10.1371/journal.pone.0153672

**Published:** 2016-04-13

**Authors:** Idil Yet, Cristina Menni, So-Youn Shin, Massimo Mangino, Nicole Soranzo, Jerzy Adamski, Karsten Suhre, Tim D. Spector, Gabi Kastenmüller, Jordana T. Bell

**Affiliations:** 1 Department of Twin Research and Genetic Epidemiology, King's College London, London, United Kingdom; 2 Department of Human Genetics, Wellcome Trust Sanger Institute, Hinxton, United Kingdom; 3 Medical Research Council (MRC) Integrative Epidemiology Unit, School of Social and Community Medicine, University of Bristol, Bristol, United Kingdom; 4 Institute of Bioinformatics and Systems Biology, Helmholtz Zentrum München, Neuherberg, Germany; 5 Weill Cornell Medical College Qatar, Doha, Qatar; University of Bristol, UNITED KINGDOM

## Abstract

Metabolomic profiling is a powerful approach to characterize human metabolism and help understand common disease risk. Although multiple high-throughput technologies have been developed to assay the human metabolome, no technique is capable of capturing the entire human metabolism. Large-scale metabolomics data are being generated in multiple cohorts, but the datasets are typically profiled using different metabolomics platforms. Here, we compared analyses across two of the most frequently used metabolomic platforms, Biocrates and Metabolon, with the aim of assessing how complimentary metabolite profiles are across platforms. We profiled serum samples from 1,001 twins using both targeted (Biocrates, n = 160 metabolites) and non-targeted (Metabolon, n = 488 metabolites) mass spectrometry platforms. We compared metabolite distributions and performed genome-wide association analyses to identify shared genetic influences on metabolites across platforms. Comparison of 43 metabolites named for the same compound on both platforms indicated strong positive correlations, with few exceptions. Genome-wide association scans with high-throughput metabolic profiles were performed for each dataset and identified genetic variants at 7 loci associated with 16 unique metabolites on both platforms. The 16 metabolites showed consistent genetic associations and appear to be robustly measured across platforms. These included both metabolites named for the same compound across platforms as well as unique metabolites, of which 2 (nonanoylcarnitine (C9) [Biocrates]/Unknown metabolite X-13431 [Metabolon] and PC aa C28:1 [Biocrates]/1-stearoylglycerol [Metabolon]) are likely to represent the same or related biochemical entities. The results demonstrate the complementary nature of both platforms, and can be informative for future studies of comparative and integrative metabolomics analyses in samples profiled on different platforms.

## Introduction

Metabolomics aims to provide a comprehensive characterization of human metabolic pathways by high throughput profiling of the small molecules present in biological samples. Various metabolomics platforms have been established to date, based on mass spectrometry (MS) or nuclear magnetic resonance (NMR) technology. However, individual platforms can differ in a number of features, including the set of metabolites quantified, the precision of quantification, and its sensitivity.

Metabolomics data have been profiled in several epidemiological cohorts [1–6], offering the potential to study the implication of metabolites in human health and disease within and across large-scale datasets. However, individual cohorts are typically profiled using different metabolomics platforms. In order to combine datasets across platforms and cohorts, there is a need to establish the extent of overlap and complementarity across metabolomics platforms.

Several previous studies have explored metabolomics datasets across multiple platforms [7–13]. For example, Suhre *et al*. [7] used multiple metabolomics platforms in a case-control study of type-2 diabetes (T2D). They profiled 100 individuals using three different metabolomics platforms to assess the potential of using metabolomic data in diabetes research by identifying metabolites that associate with diabetes. The study showed good agreement between known biomarkers of diabetes, including sugar metabolites, that could be replicated by the multiple metabolomic platform approach. Psychogios *et al*. [8] aimed to characterize the human serum metabolome by combining targeted and non-targeted NMR, GC-MS and LC-MS methods to identify a comprehensive set of metabolites commonly detected and quantified in human serum samples. They reported good agreement between the measured concentrations of NMR and GC-MS. Nicholson *et al*. [12] and Raffler *et al*. [13] studied genetic influences on NMR derived urine and plasma metabolites along with MS derived metabolites. However, these studies did not extensively compare the genome-wide findings for metabolite profiles from the same individuals to assess whether associations from datasets across platforms overlap.

In our study, we focus on the comparison of metabolites that are quantified on targeted and non-targeted mass spectrometry platforms and on the comparison of their genetic associations across platforms. Two of the most commonly used high-throughput techniques in large cohort studies apply either a targeted approach using the Biocrates platform or a non-targeted approach using the Metabolon platform. The Biocrates method is a quantitative screen of selected metabolites detected with multiple reaction monitoring, neutral loss and precursor ion scans. Metabolites are then quantified by comparison to structurally similar molecules labelled with stable isotopes added to the samples in defined concentrations as internal standards. In contrast, a non-targeted approach such as Metabolon determines relative concentrations of as many metabolites as possible without using internal standards for absolute quantification. The Biocrates AbsoluteIDQ p150 kits have been applied to quantify a targeted set of 163 metabolites, focusing predominantly on lipids. On the other hand, Metabolon has used ultra high-performance liquid chromatography coupled to tandem mass spectrometry (UHPLC/MS/MS) and gas chromatography coupled to mass spectrometry (GC/MS) for measuring around 500 metabolites from all major pathways including lipids, amino-acids, xenobiotics, and unknown compounds. Although, the methods for quantifying metabolites are distinct, there is an overlap of 43 metabolites that are measured by both platforms. Both platforms focus on different pathways, and combining datasets across platforms can help uncover a wide spectrum of complementary metabolites.

In this study we aimed to compare the Biocrates and Metabolon platforms by integrating human genetic data in a genome-wide association study design. Genome-wide association studies of metabolomic profiles (mGWAS) provide a new approach to evaluate the impact of genetic variation on human metabolism and its indirect link to complex diseases [12,14–20]. A number of studies have reported strong associations between human genetic variants and metabolites from both targeted and non-targeted metabolomics platforms [9,21–25]. The results have identified biologically meaningful associations and in some cases have been used to predict unknown gene function or metabolite identity. We propose to use mGWAS as a method of assessing biologically relevant overlap and complementarity between platforms, as the results could identify metabolites that capture shared biological processes through harmonization of two metabolomics platforms.

We present mGWAS results of metabolites measured across the two platforms in the same set of serum samples from 1,001 individuals. Our aim was to identify metabolites across platforms with consistent genetic associations, which therefore appear stable and robust across multiple platforms. The results can be used to assess how well different metabolomics profiling methods identify identical molecules, to identify metabolites under shared genetic influences, and ultimately to help identify potential metabolites for which data could be combined in future studies. Our approach shows that the different technologies are predominantly complementary in the type and set of metabolites covered.

## Materials and Methods

### Ethics Statement

The study was approved by St. Thomas’ Hospital Research Ethics Committee, and all twins provided informed written consent.

### Study Population and Sample collection

The 1,001 participants in this study were selected from the TwinsUK cohort [26]. Tests and questionnaires applied to the participants have been described elsewhere [3]. The sample consisted of 79 monozygotic (MZ) twin pairs, 215 dizygotic (DZ) twin pairs, and 413 unrelated individuals. TwinsUK blood serum samples for Metabolon and Biocrates platform were obtained after at least 6 hour of fasting and were inverted three times, followed by 40 min resting at 4°C to obtain complete coagulation. The samples were then centrifuged for 10 min at 2,000*g*. Serum was removed from the centrifuged tubes as the top yellow translucent layer of liquid. Four aliquots of 1.5 ml were placed into skirted micro-centrifuge tubes and then stored in a −45°C freezer until sampling.

### Metabolomics Measurements

The same serum samples from 1,001 individuals in this study were profiled on two separate MS platforms, Biocrates and Metabolon. The Biocrates metabolomics data were generated from Helmholtz Center Munich using AbsoluteIDQ™ p150 kits provided by Biocrates Life Sciences AG (Innsbruck, Austria). The Metabolon metabolomic data were generated from Metabolon Inc. (Durham, USA). Biocrates kits were applied to quantify a targeted set of 163 stable metabolites, while Metabolon uses a non-targeted approach for measuring 499 metabolites.

The TwinsUK dataset generated on the targeted Biocrates MS platform has previously been described [22,27,28]. Sample preparation and measurements were performed as illustrated in [25]. Briefly, after centrifugation, 10 μL of serum was pipetted into a 96 well sandwich plate, which contained inserted filters holding stable isotope labeled internal standards. After drying the filters, amino acids were derivatized with 5% phenylisothiocyanate reagent (PITC). From the dried filters, metabolites and internal standards were extracted with 5 mM ammonium acetate in methanol. The solution was centrifuged and diluted with MS running solvent. Liquid handling was performed on a Hamilton Microlab STAR robot (Hamilton Bonaduz AG, Bonaduz, Switzerland). The prepared samples were analyzed by Flow Injection Analysis (FIA) tandem MS with Electrospray Ionization (ESI) on an API 4000 mass spectrometer (AB Sciex Deutschland GmbH, Darmstadt, Germany) using multiple reaction monitoring (MRM). The internal standards served as references for calculating absolute metabolite concentrations in micromolar units (μM). The Biocrates metabolomics dataset contains 163 targeted metabolites: 41 acylcarnitines [Cx:y], hydroxylacylcarnitines [C(OH)x:y] and dicarboxylacylcarnitines [Cx:y-DC]; 14 amino acids; 1 sugar; 15 sphingomyelins [SMx:y] and sphingomyelin-derivatives [SM(OH)x:y]; and 92 glycerophospholipids [PC and lysoPC]. Glycerophospholipids are differentiated with respect to the presence of ester (a) and ether (e) bonds in the glycerol moiety, where two letters (aa = diacyl, ae = acyl-alkyl) denote that two glycerol positions are bound to a fatty acid residue, while a single letter (a = acyl) indicates the presence of a single fatty acid residue. Lipid side chain composition is abbreviated as Cx:y, where x denotes the number of carbons in the side chain and y the number of double bonds. Further descriptions of the 163 Biocrates metabolites have previously been published [27–29].

The TwinsUK dataset generated on the non-targeted MS platform Metabolon has also previously been described [22,23,30] and in this study we report results from a subset of 1,001 individuals from the overall sample. Sample preparation, measurement and metabolite identification have been performed as illustrated in [31,32]. Briefly, metabolites were extracted from 100 μl serum with 400 μl methanol (containing recovery standards) in a 96- deep well plate format. After centrifugation, the supernatant was split into four aliquots per sample: two for two separate ultra-high performance liquid chromatography/MS (UHPLC/MS) injections, one for gas chromatography/MS (GC/MS) injection, and one reserve aliquot. After drying, the aliquots were reconstituted with 0.1% formic acid, for LC/MS positive ion mode, and with 6.5 mM ammonium bicarbonate pH 8.0 for negative ion mode. The GC/MS aliquots were derivatized for 1 h at 60°C with N, O-bistrimethylsilyl-trifluoroacetamide in a solvent mixture of acetonitrile:dichloromethane: cyclohexane (5:4:1), containing 5% triethylamine and retention time markers. Pipetting was performed on a Hamilton MLStar (Hamilton Company, Salt Lake City, UT, USA) robotics system. UHPLC/MS analysis was performed on an LTQ mass spectrometer (Thermo Fisher Scientific Inc., Waltham, MA, USA) equipped with a Waters Acquity UPLC system (Waters Corporation, Milford, MA, USA). Full scan mass spectra (99–1000 m/z) and data dependent MS/MS scans with dynamic exclusion were recorded in turns. GC/MS analysis was done on a Thermo-Finnigan Trace DSQ fast-scanning single-quadrupole mass spectrometer, equipped with a 20 m x 0.18 mm GC column with 0.18 μm film phase consisting of 5% phenyldimethylsilicone. Mass spectra in a scan range from 50–750 m/z were recorded. For metabolite identification, the generated spectral data were compared against an in-house library, which includes retention time (RT), and reference spectra from mass scan and fragmentation of molecules. For every metabolite, the raw area counts were normalized to the median value of the run day to correct for inter-day variation of the measurements. The set of 499 quantified metabolites consists of several classes of named metabolites (amino acids, acylcarnitines, sphingomyelins, glycerophospholipids, carbohydrates, vitamins, lipids, nucleotides, peptides, xenobiotics and steroids) and so-called unknown metabolites of yet unidentified chemical structure (e.g. X-11521).

### Genotyping and Imputation

Genotyping of the TwinsUK dataset was performed using a combination of Illumina arrays (HumanHap300, HumanHap610Q, 1M-Duo and 1.2MDuo). We pooled the normalized intensity data and called genotypes on the basis of the Illluminus algorithm. No calls were assigned if the most likely call had a posterior probability less than 0.95. We excluded SNPs with Hardy–Weinberg (P < 1x 10^−7^) and and with minor allele frequency < 1%. First, the sparser HumanHap300 dataset was imputed to the HumanHap610Q using phased TwinsUK HumanHap610Q haplotypes as a reference. Next, the combined panel was imputed using reference haplotypes from the HapMap2 project (rel 22, combined CEU+YRI+ASN panels). The genotyping and imputation steps for TwinsUK cohort have been described in detail previously [22,23].

### Statistical Analysis

The Biocrates and Metabolon metabolomics datasets in the 1,001 serum samples first underwent several quality control checks. Both dataset were investigated for missingness at the level of each metabolite and individual. Metabolites or individuals with missing values greater than 15% were excluded from further analysis. Outliers at more than 4 standard deviations from the mean of each metabolite were excluded. In total, 11 metabolites were removed from the Metabolon dataset (out of 499 total) and 3 metabolite were removed from Biocrates dataset (out of 163 total)(S1 Table). We next performed Principal Component Analysis (PCA) on the metabolomics profiles in each dataset and compared the first 5 principal components with potential covariates to assess which variables should be included in downstream analyses. Sex, age and BMI were nominally associated with at least 1 principal component and as a result were included as covariates in the downstream analyses.

Altogether, there were 488 (Metabolon) and 160 (Biocrates) metabolites that passed quality control checks, and of these 43 metabolites overlapped, that is, were assigned to be the same molecule by both detection technologies. In the case of lyso-phosphatidylcholines (lysoPCs), the two platforms actually measure not the same but similar molecules: while Metabolon can differentiate between the position of the fatty acid residue on the glycerol backbone (e.g. 1-arachidonoylglycerophosphocholine and 2-arachidonoylglycerophosphocholine), Biocrates measures the sum concentration of both molecules (e.g. lysoPC aa C20:4). Pearson correlation was computed between the metabolite profiles across platforms to assess similarities in metabolite measurements. Several approaches can be used to normalize metabolite data, for example, log transformation [23], inverse normalization [19], and others. Here we used log transformation (base 10) after quantile normalization since test of normality showed that in most cases the normalized concentrations were closer to a normal distribution than the untransformed values. Hierarchical clustering of the metabolites was performed using the complete linkage method that finds similar clusters. All metabolomics quality control analyses were performed using R 3.0.1 (*r-project*.*org*).

Initial platform comparison focused on correlation analysis of the 43 metabolites across the two platforms. Follow up platform comparisons included genetic data for biological interpretation of platform overlap. Here, we first calculated twin-based heritability of the metabolite profiles to identify genetically stable and robust profiles across platforms [33]. Second, we used a GWAS approach to identify specific genetic variants that were associated with metabolite levels across platforms.

Heritability was computed for 43 metabolites by comparing metabolite profiles in MZ and DZ twin pairs using the ACE (additive genetic effects (A), common environment (C), and unique environment (E)) model in the OpenMx software [34]. The goal of these analyses was to establish the influence of genetic effects on metabolite profiles, to identify stable genetically determined metabolites, and to relate the results to the mGWAS findings.

To further assess evidence for genetic impacts on metabolites, we performed mGWAS analyses aiming to identify metabolite Quantitative Trait Loci (mQTLs), that is, genetic loci at which genetic variants associated with metabolite levels. We performed mGWAS using GEMMA [35], which implements a genome-wide efficient mixed model association algorithm specifically suitable for the analysis of related individuals, and provides exact P-values from linear mixed models. GEMMA tests for association between each metabolite and each SNP, using one of three commonly used test statistics (the Wald test, the likelihood ratio or score). Here we report all three statistics, but consider the Wald test when setting thresholds. We used Bonferroni correction to account for multiple testing, resulting in genome-wide significance thresholds of P = 3x10^-10^ for Biocrates and P = 1x10^-10^ for Metabolon. The mGWAS analyses were performed using common SNPs, but both common and rare genetic variants can influence metabolite profiles. The heritability results identify metabolites that are genetically determined, and these effects can be due to either common or rare genetic variants. Therefore some of the heritability effects, especially those underlying rare variants, may not be captured by the mGWAS results.

## Results

### Platform comparison: correlation and heritability of metabolites profiles

**Fig 1:**
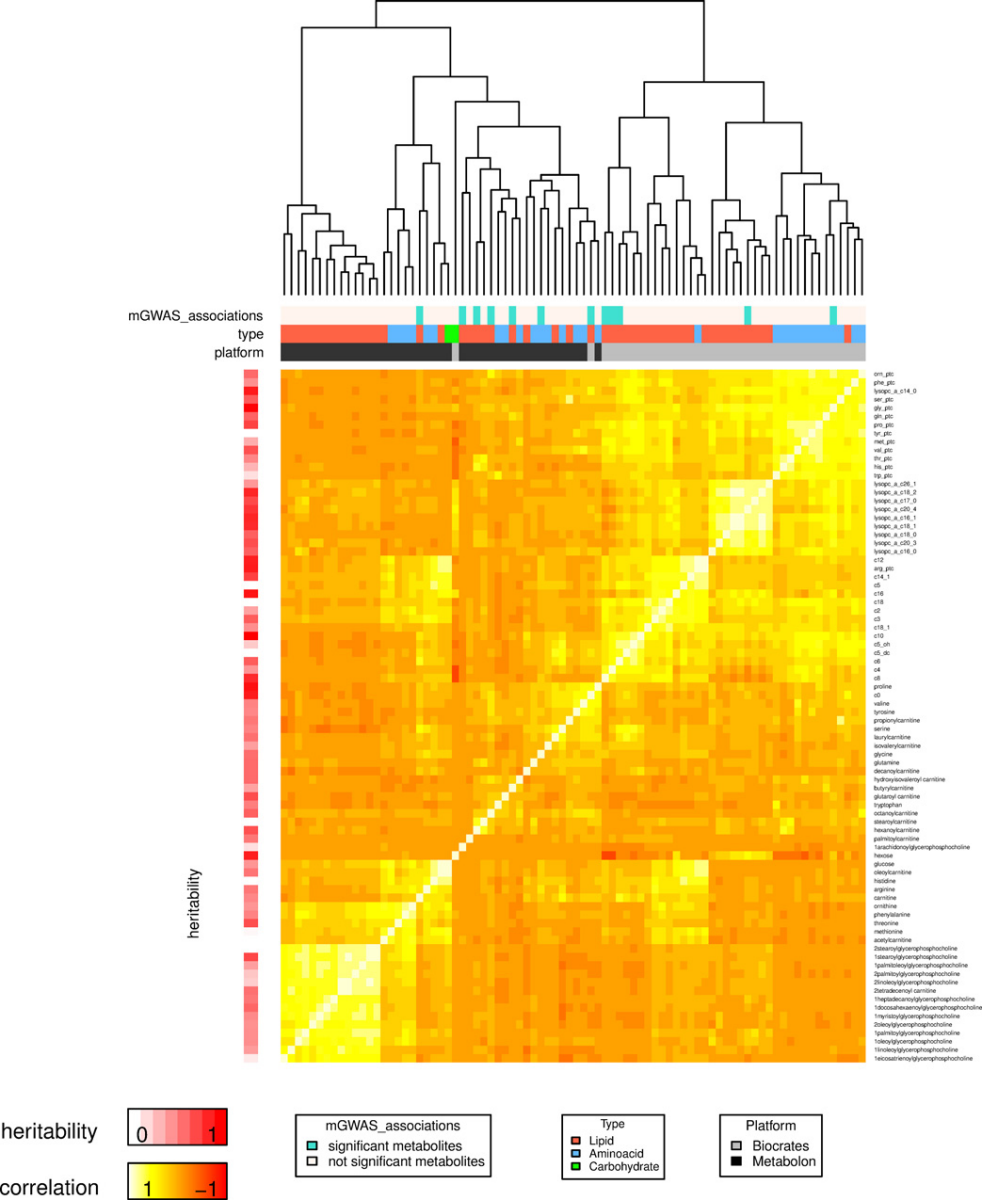
Hierarchical cluster of the correlation across 43 overlapping metabolites from both platforms. Upper colour bars represent metabolites with mGWAS results, metabolite type, and metabolite platform. The left colour bar represents the heritability of the metabolite from red (high) to white (low).

Following quality control assessment, there were 488 (Metabolon) and 160 (Biocrates) metabolites available for analysis in serum samples from 1,001 individuals. Of these, 43 were designated as overlapping molecule by both platforms (S2 Table). Comparisons of the 43 metabolites showed a mean correlation coefficient (r) of 0.44 with a maximum correlation for octanoylcarnitine (r = 0.92), minimum correlation for 1-docosahexaenoylglycerophosphocholine (r = 0), and weak correlations (0<r<0.2) for 7 metabolites (S2 Table), which included lipids and an amino acid. Using hierarchical clustering of the correlation matrix, we observed that the metabolites tend to cluster first within platform, and then within type of the metabolite (Fig 1). One clear exception is hexose (Biocrates), which clusters with glucose in the Metabolon cluster, as expected. A second exception is carnitine C0 (Biocrates), which clusters near proline, valine, tyrosine, and propionylcarnitine in the Metabolon cluster. Additionally, we calculated the correlation between the 43 metabolites and all remaining metabolites on both alternative platforms. We observed that the resulting correlations were overall much lower, and only two pairs of metabolites across platforms had a mean correlation of 0.44 or greater; these included octanoylcarnitine C8 (Biocrates) and the unknown metabolite X-11521 (Metabolon), and laurylcarnitine C12 (Biocrates) and the unknown metabolite X-18739 (Metabolon).

Because the 1,001 individuals included twins, we were able to calculate twin-based heritability estimates of the metabolite profiles, focusing on the 43 overlapping metabolites (S2 Table). Of the 43 metabolites, 37 (Biocrates) and 34 (Metabolon) were at least moderately heritable in twins (h^2^>0.2). There were 29 metabolites with evidence for heritability on both platforms (h^2^ ranging from 0.29 to 0.72, S2 Table). Of these, the 9 most heritable profiles were observed for 6 lipids (h^2^: 0.4 to 0.72) and 3 amino acids (h^2^: 0.42 to 0.7), indicating that these are stable profiles and highly likely to be under genetic influence.

### mGWAS results: overlapping and complementary mQTLs

In total, 488 and 160 metabolites were tested separately on the Metabolon and Biocrates platforms in two mGWAS analyses. All genome-wide significant association results are reported at a stringent Bonferroni cut-off: P = 1×10^−10^ (5×10^−8^/488) for Metabolon and P = 3×10^−10^ (5×10^−8^/160) for Biocrates. Additionally a relaxed threshold for genome-wide association (5×10^−8^) was used to evaluate whether a mGWAS finding on one platform was replicated on the other platform (S3 Table). Additionally, we provide all result pairs where metabolites on both platforms surpassed evidence for genetic association at P = 5x10e-8 (S4 Table)).

In total, 61 genome-wide significant metabolite associations were identified at 26 independent loci: 42 metabolites were associated with 25 loci on the Metabolon platform, and 19 metabolites were associated with 8 loci on the Biocrates platform (Table 1). Of the 26 independent loci, genome-wide significant metabolite associations at 7 loci were identified on both platforms. There were 19 loci that had associations only with metabolites from one platform (18 loci in Metabolon and 1 locus in Biocrates).

**Table 1:** Genome-wide significant mGWAS results.

	Loci^a^	All associated metabolites	Associated metabolites from set of 43 overlapping metabolites^b^
Metabolon (M)	25	42	6
Biocrates (B)	8	19	7
Overlap	7	22(13M + 9B)	6
Total	26	61 (35M+12B+7M&B+7B&M)	13

^a^Unique loci

^b^Metabolites with genome-wide significant mGWAS results from the set of 43 matching metabolites only. In all cases the reciprocal platform mGWAS result surpassed nominal significance with the same direction of association.

### Overlapping mQTLs: genetic associations identified on both platforms

Associations at 7 independent loci were identified in both platforms, namely with SNPs in the regions of the *ACADM*, *ACADL*, *CPS1*, *SLC16A9*, *FADS1*, *ACADS* and *SGPP1* genes (Table 2). The 7 loci associate with 22 metabolites in total: 9 metabolites from Biocrates and 13 metabolites from Metabolon.

**Table 2:** mGWAS results at 7 loci associated with metabolites in both platforms.

Locus	Chr	Position	SNP	Biocrates (P = 3×10^−10^)	Metabolon(P = 1×10^−10^)
***ACADM***	1	75,879,263	rs211718	-	*X-11421(3.8×10^−8^)
		75,934,477	rs4949874	C6(4.1×10^−11^)	Hexanoylcarnitine(1.6×10^−13^)
		76,103,908	rs2172507	*C8(2.4×10^−8^)	Octanoylcarnitine(4.8×10^−11^)
***ACADL***	2	210,764,902	rs7601356	C9(9.7×10^−38^)	-
		210,715,532	rs12612970	-	X-13431(3.5×10^−25^)
***CPS1***	2	211,316,624	rs4673553	Glycine(5.3×10^−17^)	Glycine(7.1×10^−27^)
		211,316,624	rs4673553	-	X-08988(1.6×10^−11^)
***SLC16A9***	10	61,139,544	rs1171614	C0(4.6x10^-12^)	-
		61,137,188	rs1171617	-	Carnitine(2.3×10^−13^)
***FADS1***	11	61,326,406	rs174546	*PC ae C42:5(1.9×10^−8^)	*1-Linoleoylglycerophosphoethanolamine(1.2×10^−8^)
		61,327,359	rs174547	lysoPC a C20:4(2×10^−14^)	*1-Arachidonoylglycerophosphocholine(2.9×10^−10^)
		61,327,359	rs174547	-	*Arachidonate(20:4n6)(5.5×10^−10^)
***ACADS***	12	119,644,998	rs2066938	C4(2.9×10^−44^)	Butyrylcarnitine(1.8×10^−114^)
***SGPP1***	14	63,305,309	rs7157785	*PC aa C28:1(3.8×10^−8^)	1-Stearoylglycerol(2.8×10^−14^)
		63,305,309	rs7157785	-	*X-10510(1.4×10^−9^)

*Shown at a relaxed genome-wide cut-off (5x10^-8^)

Of the 22 associated metabolites, 6 metabolites associated with 5 loci were named for the overlapping compound on both platforms. These included C6 (Biocrates, P = 4.1×10^−11^) = hexanoylcarnitine (Metabolon, P = 1.6×10^−13^), C8 (Biocrates, P = 2.4×10^−8^) = octanoylcarnitine (Metabolon, P = 4.8×10^−11^), glycine (Biocrates, P = 5.3×10^−17^) = glycine (Metabolon, P = 7.1×10^−27^), C0 (Biocrates, P = 4.6×10^−12^) = carnitine (Metabolon, P = 2.3×10^−13^), C4 (Biocrates, P = 2.9×10^−44^) = butyrylcarnitine (Metabolon, P = 1.75×10^−114^), and lysoPC a C20:4 (Biocrates, P = 2×10^−14^) = 1-arachidonoylglycerophosphocholine (Metabolon, P = 2.9×10^−10^), as designated by Biocrates and Metabolon, respectively. For three of the 5 loci with smatching named metabolites, there were also associations with other metabolites, which do not necessarily match across platforms (Table 2).

In one case genetic variants in locus *ACADL* were associated with both a Biocrates metabolite C9 (P = 9.7×10^−38^) and an unknown Metabolon metabolite (X-13431 (P = 3.5×10^−25^)), which were recently shown to be identical molecules [36]. The mean correlation coefficient between these metabolites across platforms was moderate (r = 0.54, Fig 1).

In one case, metabolite associations with genetic variants at the *SGPP1* locus did not match in name for PC aa C28:1 (Biocrates) and 1-stearoylglycerol (Metabolon) (Table 2). The mean correlation coefficient between these metabolites across platforms is moderate (r = 0.42, Fig 1). Both of these are lipid metabolites, and could share the C18:0 fatty acid chain.

### Complementary mQTLs: genetic associations identified in only one platform

There were 19 loci that had associations only with metabolites from one platform (18 loci in Metabolon and 1 locus in Biocrates) and these all were associated with metabolites that were not measured in the other platform (S3 Table).

The 18 Metabolon-specific mGWAS results included associations with 29 metabolites. Of these 29 metabolites, 17 were unknowns, 4 were lipids and 3 were amino acids and these were not included in Biocrates, considering that Biocrates consists mostly of lipids and amino acids. The 5 remaining metabolites were 2 drugs, a carbohydrate, a nucleotide, and a peptide.

There was only 1 locus (*DYNC1H1*) where genetic variants showed genome-wide significant mGWAS results on the Biocrates platform only with 4 metabolites, and in all 4 cases these were with lipids that Metabolon did not measure.

## Discussion

Our study is a bi-platform metabolite comparison using mGWAS with the objective of identifying metabolites measured on more than one platform where signals overlap and may be combined in future studies, for example for replication analysis. The key results identified 7 loci showing robust genetic associations with metabolites on both platforms. These results were also predominantly consistent with recent reported mGWAS [22,23,30,37], some of which are based on results from extended cohorts that include the samples used in the current analysis. Thus, for 6 of the 7 loci (*ACADM*, *ACADL*, *CPS1*, *SLC16A9*, *FADS1*, *ACADS*), previous mGWAS reported associations with the same Metabolon metabolite either as a single metabolite or as part of a metabolite ratio [23,30]. In contrast, *SGPP1* harboured an mQTL with the Metabolon metabolite ratio (X-08402/cholesterol), and the single metabolites X-08402 and X-10510 in Shin *et al*. [28], while here we report associations with 1-stearoylglycerol and X-10510.

Of the metabolites associated with the 7 loci, 5 metabolites (Biocrates C8, C6, C0, C4, and glycine) had at least moderate heritability (h^2^>0.26) and correlation (>0.38) on both platforms, confirming that these profiles are stable and reproducible across platforms. Interestingly 1 matching metabolite, lysoPC a C20:4 [Biocrates] / 1—arachidonoylglycerophosphocholine [Metabolon], showed low heritability in one platform (0.09 in Metabolon and 0.59 in Biocrates platform) and showed relatively low correlation (r = 0.29) across platforms, but was still identified to associate with the same locus from both platforms at genome-wide significance. This observation may be due to the difference in the measured compounds between the two platforms: while Metabolon specifically quantifies the lysoPC with the 20:4 fatty acid chain at *sn1* position of the glycerol backbone (lysoPC(20:4/0:0), Biocrates does not distinguish between the lysoPCs with fatty acid chains at *sn1* and *sn2* positions and only quantifies the sum concentration of the two forms (lysoPC(20:4/0:0 and lysoPC(0:0/20:4). Moreover, the quality of measurement differs for various lipids between the targeted Biocrates and the non-targeted Metabolon platform, which might also cause lower correlation between the corresponding matching metabolites. Notably, despite those differences inherent in the platforms both profiles give a robust signal of genetic association for *FADS1*.

Further comparison of the GWAS results across platforms shows that genetic variants at 5 of the 7 loci (*ACADM*, *CPS1*, *SLC16A9*, *FADS1*, *ACADS*) were associated with metabolites that were named for the overlapping compound. However, genetic variants at the *ACADL* and *SGPP1* loci only associate with non-overlapping metabolites or unknown metabolites from the Metabolon platform. In these cases, our results can be used to inform the function of unknown metabolites or identify metabolites that belong to the same or related biological pathways. For example, variants in the *ACADL* locus associated with the C9 Biocrates metabolite and also with the unknown X-13431 Metabolon metabolite, which were recently reported to be the same molecule [34]. When we explored the results for similar association patterns, we observed that Metabolon metabolites X-10510 and 1-stearoylglycerol shared mQTL findings within the same locus (*SGGP1*) as the Biocrates metabolite PC aa C28:1. These results suggest a link between the molecules, where the more specific Metabolon lipid chain length can hint that the PC aa C28:1 association is possibly driven by the involvement of a 18:0 lipid chain. Alternatively, the *SGGP1* genetic variant (rs7157785) has also been associated with sphingomyelin 14:0 in a separate study [35]. Our platform does not include this metabolite, but X-10510 may be also related to this sphingolipid pathway. This assumption is further supported by high partial correlation between X-10510 and other Metabolon sphingolipid molecules and genetic associations to a second sphingolipid related gene in Shin *et al*. [30].

We next explored the 43 overlapping metabolites on both platforms for consistencies and potential inconsistencies across platform signals beyond their association results. As expected, the mean correlation between the 43 matching metabolites (r = 0.44) is higher than the mean correlation with all metabolites between the two platforms (r = 0.17). Exceptions include correlations of Biocrates metabolites with Metabolon metabolites of yet unknown chemical identity. In these cases, the high correlation could indicate matching metabolites or biochemically related metabolites and might thus again assist in the identification of unknown metabolites.

Four lyso-phosphatidylcholine metabolites (lysoPC a C16:0, lysoPC a C18:0, lysoPC a C18:1, lysoPC a C18:2) from the Biocrates platforms had overlapping metabolites on the Metabolon platform, but neither contained matching mQTLs nor showed high heritability or correlation. We conclude that in this instance the two platforms are likely measuring distinct signals that cannot be combined or this may be due to a relatively lower quality of measurement for these lipids on the Metabolon platform.

We applied a combination of correlation, heritability and genotypic analyses to bring together the comparison of data from different metabolomics platforms. Our approach identified genetic associations at 7 loci with pairs of metabolites measured on the two platforms that were named for the same compound, were highly correlated and heritable, therefore suggesting that in these cases Biocrates and Metabolon signals overlap. In contrast, 9 pairs of known metabolites that are not named for the same compound across platforms, but exhibit similar levels of correlation and heritability, showed no overlapping genetic associations. The two platforms are designed to focus on different metabolites, and these findings can inform on platform-specific metabolites. Ultimately, combining metabolomics profiles across platforms is more informative than single-platform analysis because platforms are complementary. It is not possible to assay the entire metabolome with one platform due to large differences in the physiochemical properties of the different metabolites (e.g. lipophilic and hydrophilic metabolites).

In summary, we identified genetic associations at 7 loci with metabolite profiles from both the Biocrates and Metabolon platforms. Our results provide new information about potential shared pathways, as well as distinct metabolite profiles, and their genetic determinants, clarifying unknown metabolites. Our study demonstrates the complementary nature of both targeted and non-targeted MS platforms and can help future studies to explore combining datasets across platforms, especially for replication of metabolite hits when datasets are profiled on different platforms. The findings can help guide further research into the sources of inconsistency and variation in the comparison of metabolite results profiled from differing platforms.

## Supporting Information

S1 Table Metabolites removed from analysis at Quality Control. (XLSX)

S2 Table Metabolon and Biocrates platform comparison at 43 overlapping metabolites including correlation, heritability and peak mGWAS results. (XLS)

S3 Table mGWAS results for Biocrates and Metabolon platforms. (XLSX)

S4 Table mGWAS Results for Biocrates and Metabolon with relaxed threshold (5e-8). (XLSX)
